# Care mobilities and associated contexts of hospital-based informal caregiving in Nigeria: Towards an explanatory framework

**DOI:** 10.1371/journal.pone.0327198

**Published:** 2025-07-31

**Authors:** Kudus Oluwatoyin Adebayo, Oluwaseyi Dolapo Somefun, Mofeyisara Omobowale, Rukayat Usman

**Affiliations:** 1 Institute of African Studies, University of Ibadan, Nigeria; 2 School of Public Health, University of The Witwatersrand, South Africa; 3 School of Public Health, University of the Western Cape, Cape Town, South Africa; 4 Institute of Child Health, College of Medicine, University of Ibadan, Nigeria; 5 Department of Sociology, University of Ibadan, Nigeria; Sunu Sante Consulting, SENEGAL

## Abstract

Hospital-based informal caregiving in Nigeria is shaped by care mobilities and contextual factors such as policy contradictions and normative care philosophies. This study explores how these factors influence caregiving practices in a Nigerian tertiary health facility. Using a qualitative approach, data were gathered through interviews and observations, involving 75 participants, including 36 in-depth interviews with caregivers and inpatients, and 39 key informant interviews with staff like nurses, doctors, security guards, and ad-hoc caregivers. Findings showed that many informal caregivers traveled long distances to assist hospitalized relatives, often “hanging around” the hospital and engaging in micro-mobilities, such as running errands. Geographical distance, policy contradictions, and the financial costs of hospitalization significantly affected caregiving dynamics. Care mobilities caregivers moving within the hospital environment emerged as a critical aspect of the caregiving process. Understanding these mobilities and how they intersect with contextual factors is essential to improving caregiving experiences. The study highlights the need for policies that support informal caregivers and enhance patient outcomes, especially in terms of reducing the burdens caregivers face due to long travel distances, hospital policies, and financial challenges.

## Introduction

How do care mobilities and other contextual factors influence the dynamics of hospital-based informal caregiving (HIC)? Globally, extensive research has been conducted on the roles and practices of informal caregivers (ICs) within both formal healthcare systems and general care provision settings [1–4]. In Africa, and various low-and-middle income settings, ICs play crucial roles in supporting relatives and friends experiencing ill-health. Their lived experiences encompass a range of tasks and responsibilities, extensively documented in scholarly literature [5,6]. The burdens and challenges faced by ICs can be particularly pronounced when providing care to hospitalized individuals [7,8].

However, extant studies have done little to explore the contextual factors influencing informal caregiving in hospital settings. This article aims to address this gap by investigating the role of care mobilities and other contextual factors that drive the involvement of relatives in hospital-based informal caregiving (HIC). Specifically, this study examines how relatives of inpatients engage in HIC, providing informal care while remaining in or around a healthcare facility. For the purpose of this study, migrating informal caregivers are a sub-population of ICs who travel far away from their place of residence to support the care of inpatients while stationed in/around health facilities.

Informal caregiving is an essential component of the global healthcare system [9]. The World Health Organisation (WHO) describes an IC as “any person without formal health training who is not employed by the hospital facility and is onsite in the capacity as ‘carer’ or ‘guardian’ of a person known to them who is admitted to the facility as a patient” [10]. Informal caregivers (ICs) play a vital role in supporting patients, particularly in Africa, where the majority are women [11], they are integral to Universal Health Coverage efforts, providing invaluable labor to health systems [12].

In numerous African settings, ICs persist in their supportive roles, influenced by a multitude of complex and interconnected factors [13]. These factors include both intrinsic (dispositional) and extrinsic (external) elements. Intrinsic factors stem from the caregivers’ internal motivations, driven further by personal ability, values and beliefs [1]. Extrinsic factors arise from external influences such as familial obligations, cultural influences, severity of ailment of the care recipient, poverty and health systems dysfunctions. For example, cultural precepts prescribe a feeling of obligation to act as an IC as entrenched in the principles of reciprocity, responsibility and selfless affection [2,14].

ICs face significant challenges and burdens, with studies consistently reporting high levels of caregiver burden, vulnerability to depression, and adverse impacts on physical, emotional, and general health [14–16]. These challenges are exacerbated by factors such as recurrent hospitalization, treatment uncertainties, prolonged caregiving, and reduced patient capabilities [17]. Additionally, the economic burden is considerable, particularly for young caregivers who are in their most economically active years [17].

Providing informal care to inpatients presents additional challenges. The hospital environment’s strict rules may conflict with the consistent presence and involvement of relatives [18,19]. ICs provide diverse assistance, including personal and medical care, often performing specialized tasks such as handling samples and assisting with mobility [20,21]. These responsibilities can adversely affect ICs’ social, financial, and health aspects [22]. Furthermore, inadequate funding, infrastructure deficits, staff shortages, and a lack of patient-centered care exacerbate the challenges within the hospitalization structure [23, 24].

In Low- and middle-income countries like Nigeria, where familial networks often serve as the primary source of support for individuals facing health challenges, informal caregivers (ICs) play a vital role in complementing formal healthcare services. However, the role of ICs within hospital settings remains underexplored, particularly regarding the influence of care mobilities, the movement of patients, caregivers, and resources within and across healthcare systems on HIC practices.

Nigeria, the most populous nation in Africa, is divided into six geo-political zones: South East, South West, South South, North East, North Central, and North West, and operates as a political federation with 36 states comprising multiple ethnic groups. Although Nigeria’s health system has developed over time, it remains fragile, inequitable, and inefficient. It consists of a complex mixed system where private hospitals operate in a free market, and public hospitals are under government control. The private sector delivers approximately 60% of healthcare services, whereas the public sector accounts for 40%. However, the public health sector is nearing collapse due to inefficiencies, poor infrastructure, and insufficient resources [25].

Responsibility for healthcare is divided among local, state, and federal governments, which oversee primary, secondary, and tertiary care, respectively. However, there is a lack of coordination among these levels due to overlapping responsibilities. The primary healthcare (PHC) system, meant to be the backbone of Nigeria’s health system, has struggled to provide essential services due to inadequate funding, deteriorating infrastructure, poor governance, subpar service delivery, and low performance among health workers [25,26]. Patients often have to pay out-of-pocket for medications and provide their own meals during hospital stays due to inadequate funding and resource allocation within the healthcare system [25]. This financial burden is compounded by a severe shortage of healthcare workers, which results in high patient-to-doctor ratios and limited access to specialized care, particularly in rural areas [27]. Rural-urban disparities further exacerbate these challenges, with urban centers typically having better-equipped facilities and more healthcare professionals compared to rural regions. These systemic issues necessitate the reliance on informal caregivers (ICs) who travel significant distances to support their hospitalized relatives, often becoming “resident caregivers” within hospital premises. This reliance is influenced by cultural expectations and the inadequacies of the formal healthcare system, making the role of ICs crucial yet underexplored in the context of hospital-based informal caregiving (HIC). Understanding the healthcare system’s dynamics is essential to comprehensively studying how care mobilities and other contextual factors shape the practices and challenges of HIC in Nigeria.

Despite the prevalence of informal caregiving, explanations for the occurrence and persistence of hospital-based informal caregiving are often limited, particularly regarding the critical significance of informal care-related mobilities. Care mobilities play a crucial role in providing a comprehensive understanding of informal caregiving in hospitalization situations. These mobilities entail leaving one’s home and traveling to support family members who need to access healthcare [28,29].

On one hand, individuals residing close to healthcare facilities are less likely to require hospitalization compared to those living farther away [30]. Conversely, referred patients often seek tertiary healthcare outside their state or province of residence [31]. Consequently, informal caregivers (ICs) engage in trans-localism by undertaking intra- and inter-district/state level travels to assist sick relatives in the hospital [28,32]. Due to the costs of daily commuting and their economic circumstances, ICs often travel long distances and become “resident caregivers” by residing within hospital premises [28]. Understanding the role of care mobilities in the practice of hospital-based informal caregiving (HIC) is therefore essential for comprehensively studying informal caregiving dynamics. Although hospital-based informal caregiving is widespread, the underlying ‘care mobilities’ from trans-local journeys to extended on-site stays remain largely unexplored, revealing critical social, economic, and emotional forces that shape both the practice and sustainability of this form of care.

In this paper, our objective is to develop a comprehensive understanding of hospital-based informal caregiving (HIC) in low- and middle-income countries, drawing from data collected at a Nigerian tertiary health facility. We aim to explore the significance of care mobilities in shaping HIC practices. Specifically, we seek to demonstrate how care mobilities, alongside other dynamics within the hospital and healthcare systems, contribute to the conditions conducive to HIC. The subsequent sections of this paper outline the methodology employed in our study, present the findings, and engage in a discussion and analysis of these findings. Finally, we conclude with insights gleaned from our research.

## Materials and methods

### Study design

The article is based on the “LEMIC Study” research designed to explore the lived experiences of internally migrating informal caregivers in a Nigerian tertiary health facility. Detailed information regarding the methodology has been published elsewhere [33]. The study relied on ethnographic methods of inquiry to collect data based on qualitative techniques, such as observation, in-depth and key informant interviews. Observation in this setting was particularly interesting because often times, the visual and olfactory senses offer first clues to the presence of an informal caregiver far from home and the open spaces where they usually slept. Observation was non-participant and was divided in two phases. The first was the “introductory phase”, meant to simply immerse the research team into the hospital environment. During this time, we simply focused on the areas where people congregated watching the actors and had informal chats with them about waiting around the hospital premises. This phase helped us develop an observation checklist, which then served as identifiers of informal caregivers themselves, objects signifying their presence or where they stayed (outside of the hospital’s formal accommodation). The second phase was further divided into diurnal and nocturnal times. Rough-sleeping around the hospital’s premises gave away those who could not go back home, while sighting these caregivers during the day revealed the day’s activities they were usually involved in. During this stage, we developed important checks. The research team recorded the time, strange objects (e.g., folded cardboard boxes in corners because they served as make-shift beds for caregivers at night), spaces traversed and related activities. These were recorded in fieldnotes. Each fieldnote was then collectively discussed, transferred from notebooks to word documents, and then coded alongside interview transcripts.

### Setting

The research was conducted in a public tertiary health facility in southwest Nigeria. The southwest region has a high concentration of tertiary health facilities, the highest level of care available in Nigeria [34]. The facility is one of the most advanced in Nigeria, provides specialist care and operates clinical and non-clinical departments. Data collection activities occurred in the “hospital community”, comprising the wards, offices, public spaces, corridors, restaurants, restrooms and other sites within and near the hospital – e.g., four varied-styled hospital-owned hostels accommodating caregivers. Although these spaces had other people in the vicinity, mainly other caregivers waiting around because they have a patient on the ward, interviews held at comfortable distances and participants spoke freely. Data collection for the research study took place from December 15, 2022, to March 30, 2023.

### Participant selection

Four categories of participants were included in the study: informal caregivers, inpatients, hospital staff, and ad-hoc/paid caregivers. Informal and ad-hoc/paid caregivers and inpatients were selected across wards with well-established inpatient services. Hospital staff were drawn based on their relevance for the research, including health workers, management staff, security guards and health assistants (see Table 1).

**Table 1:** Study population by distribution of interviewees and average interview duration.

	Study population	IDI	KII	Average interview time (in mins.)
1	Caregiver	21	–	43
2	Inpatient	15	–	33
3	Hospital staff			
	● Management	–	6	
	● Nurse	–	10	35
	● Doctor	–	5	23
	● Security guard	–	5	26
	● Health assistant (ward)	–	5	34
	● Health assistant (environment)	–	5	24
4	Ad-hoc/paid caregivers	–	3	13
	Sub-total	36	39	
	Total	75		

The participants were selected using a combination of purposive, snowball, quota, and convenience sampling methods. We used purposive sampling to identify the relevant units in the hospital. The identification of more than half of the informal caregivers was purposively conducted, while the rest were relatives who were available and are willing to participate in the study. We initially approached caregivers within and around hospital wards to inquire about their presence and subsequently introduced and invited them to voluntarily participate in the study. In the case of the 15 inpatients, we obtained necessary ethical and hospital approvals as well as patient consent before approaching and recruiting them using a purposive technique. Consent was obtained through both oral and written methods, and it was recorded; while there were no formal witnesses. Nurses identified inpatients that were fit to participate and were present during the consent process. Quota sampling was utilized to ensure diversity within the study population: most informal caregivers were women but we ensured that male caregivers were included, and maintained an age balance to capture young, middle aged and elderly caregivers. The snowball technique helped us achieve paired sampling of participants, with 9 informal caregivers providing connection with their in-patients. The technique also proved beneficial in identifying relevant staff spread across units and departments within the hospital. Additionally, convenience sampling was employed to select participants based on inclusion criteria, availability, and willingness to participate. Using a combination of sampling methods ensured the complementarity of strengths to cover for the blind-spots of a singular method. For example, snowballing’s strength in caregiver-inpatient identification helped us understand the nuances experiences of informal caregivers as shaped by the prognosis and conditions of their in-patients. This further buttressed the quota and purposive sampling methods, ensuring that diversity of conditions were covered. All interviews were conducted face-to-face.

To be eligible for inclusion in the study, hospital staff were required to be formally employed at the health facility, while ad-hoc caregivers were qualified based on their affiliation and role as paid carers. They were conveniently sampled based on scheduled appointments and availability in the hospital premises. Inpatients were included if they were currently admitted and receiving care in any of the inpatient wards, had an on-site informal caregiver who had traveled from outside the city to provide support, were able to communicate effectively, and were subjectively deemed healthy enough to participate based on assessment and permission from the nurse on duty.

Furthermore, informal caregivers were eligible if their usual residence was located outside the city where the hospital is situated, they were attending to an inpatient who had been admitted for three days or more, resided or had resided in or around the healthcare facility during data collection, were of any gender, and aged 13 and above. In total, 75 participants were interviewed. (see Table 1).

### Interviews

Seventy-five (75) participants were interviewed, including 36 IDIs (caregivers, 21; inpatients, 15) and 39 KIIs (management staff, 6; nurses, 10; doctors, 5; security guards, 5; health assistants, 10, and; ad-hoc/paid caregivers, 3). We documented the background information of caregivers and discussed issues related to their motivations, the health situation/diagnosis of their care recipients, health-seeking related mobilities/migration, experiences of hospitalisation and relationship with hospital staff and co-caregivers. We found ICs who participated in the study because they considered it as a means to pour out their hearts and gain some solace while at it. Some others saw research as a means of disseminating their challenges until it catches the attention of the right quarters, who can help them mitigate their challenges. However, some other ICs were not interested in the study because they considered themselves too busy “running up and down”. The research team mostly met the ICs while they were waiting to be allowed in the wards, around temporary stay areas or moving frantically around the hospital premises. In these settings, some ICs throw some rhetoric questions at us, such as “if you were the one, wouldn’t you think that is sheer wickedness?” “How would you react if it was your family on the bed”? While we tactfully remained silent, or calmly moved our heads to show concern, or responded with “hmm” (in most Nigerian contexts, this is a subtle exclamation which shows ones attentiveness in a conversation), these sort of questions made us reflect on our positions and conduct interviews and relate sympathetically with participants. Also, encounters with the in-patients and their caregivers was usually an emotive experience. We noticed the pensive combination of stress, gratitude, despair and sometimes anger (at hospital management) between informal caregivers and their patients. During deeply emotive times like tearful moments, we expressed sympathy, and paused the conversation until the participants felt like carrying on. We acknowledge that it was difficult conducting interviews in these states, especially when one reflects on our limitation of any immediate powers to make their conditions better. We have presented a deeper reflexive statement elsewhere as it relates to the broader study from which this article emerged [35].

Furthermore, inpatients were asked to tell us about the situation of the informal caregivers supporting them in the hospital, including sharing the extent of their familiarity with what these caregivers go through, the roles they play, and their perception of how hospital staff treat caregivers. Also, we interviewed management staff, nurses, doctors, security guards, health assistants, and ad-hoc/paid caregivers, who, either by professional responsibility, job roles or affiliation, relate with caregivers. We explored with them their familiarity with informal caregivers who visit the hospital from outside the city and staying in/around the facility to support the care of relatives. We asked how they perceive and relate with informal caregivers during patient care, the contributions they think they make, how they view caregivers’ experiences of “living in the hospital”, the challenges their presence pose to patient care and the health system and suggestions for improving their condition. Security guards and health assistants, who manage hospital spaces that were important for the everyday experiences of caregivers (e.g., toilets, buildings, and open/closed hospital spaces such wards, corridors and so on), were engaged to provide further contexts that are crucial to the lives of informal caregivers in hospital setting. We wanted to understand their perceptions and everyday encounters and negotiations with the caregivers while discharging their duties. Interviews with hospital management focused on operational and governance issues surrounding the presence and treatment of informal caregivers, especially what policies and facilities are in place/or not in place to integrate their presence into the standard hospital operations.

The languages of interview were English, Yoruba and Pidgin English, a simplified, less formal form of English that is mixed with local languages and popular across West Africa. All research team members are competent users of the three. The research team are natives of the wider region (Southwest) where this research was conducted, and thus, adept at speaking and listening to Yoruba language. However, to ensure familiarity with hospital lexis in local tongue, the team went through the interview guides in Yoruba and Pidgin languages at a team training prior to commencement of fieldwork. The participants made the final decision on the language of discussion which was facilitated using interview guides written English, Yoruba and Pidgin English. We recorded all the interviews on audio recorders and took notes. Data saturation was determined when no new themes or codes emerged during the analysis of the final three interviews, indicating redundancy in responses. Two researchers independently assessed saturation and confirmed its attainment.

### Data analysis

We subjected notes from observations and field reports to ethnographic analysis by transcribing the interviews in the original language of conversation and back translated to English as applicable. This involved a process of accurate depiction of proverbs and praxis into meaningful sentences in English language. Transcription, translation, and back-translation to English was executed was outsourced to research assistants seasoned in these areas, some of which were language experts at the Department of Linguistics. Each file was cross-checked afterwards by the research team. The fieldnotes were written in English, collated together with transcripts. The transcripts were then prepared prior to exporting them into the NVivo software by relabelling them with codes pre-assigned to each participant. The thematic analysis was conducted, following the procedure outlined by Braun and Clarke [36]. Themes generated from the thematic analysis were further engaged using “…the hermeneutic circle of interpretation to assign meaning to the lived experience” [37], starting with initial immersion in the data by the lead analysts (KOA and MOO), preliminary interpretations and jointly discussed by the research team and moving back and forth in iterative manner to link each identified context of hospital-based informal caregiving to other identified contexts. This process led us to the framework we have proposed in the article. We prepared a preliminary coding structure using the deductive approach in accordance with the questions that guided the interviews with participants. Each code was defined/described to reduce ambiguity along the way. Extracted sections of the interviews across participants have been presented either as summaries or direct quotes in the findings section.

#### Reflexivity, authenticity and potential bias.

The fieldwork for this study was conducted by a research team consisting of two university-based and experienced researchers with PhD degree in Sociology (KOA) and Medical Anthropology (MOO), and three graduate students recruited as research assistants from Sociology, Public Health, and African Studies. Throughout the field activities, and during the weeks when we collected all the data for the research, the research team provided sufficient and transparent information to the research participants. We were open about our background, work and affiliations and encouraged the participants to ask questions and seek clarification, including after we might have exited the field. We also organised three sessions (one inception meeting and two photovoice sessions) where some of the participants were given a platform to express how they feel about the research project and to suggest areas where they think we should focus on. The sessions broke down barriers between the research team and the participants. The feedback received assisted the researchers to collaboratively focus the evidence gathered for dissemination purposes.

We addressed potential participants’ bias in our analysis by ensuring that we draw on the diverse voices across different categories of persons that were involved in the study. While the project seeks to centre the condition of informal caregivers, producing an explanatory framework based on the view of caregivers alone would be inadequate. By combining views across various categories of participants, we are able to general a robust explanation of why relatives of hospitalised patients perform hospital-based informal caregiving.

### Ethical considerations

An institutional ethics board reviewed and approved the study (UI/EC/22/0317) valid from 2/11/2022–1/11/2023. We also obtained two additional official permissions: first from the hospital management to collect data in the hospital community and second from the head of nursing to access the wards and speak to inpatients. Moreover, the research team adhered to ethical principles expected of studies conducted in a hospital setting, particularly informed consent, voluntariness, non-malevolence, and confidentiality. Both written and verbal informed consent were obtained from all participants. The consenting process and interviews were conducted in a language the participants preferred. For participants experiencing emotional distress, consent was obtained incrementally: researchers paused discussions, offered breaks, and revisited the process only when participants expressed readiness. Verbal consent (audio-recorded with permission) was used for those physically unable to write.

In terms of benefits, we clarified to them that, although they may not benefit directly, their participation will advance understanding on the topic and potentially shape practice and policy in the health sector. The research did not pose any serious risks to the participants but we nonetheless furnished them with information on where to go if there was ever a need to do so. No serious ethical challenge were encountered in the field, except during the photovoice process which is not part of the evidence presented in this article. Completed informed consent forms were kept at secured locations, physical and virtual, while interview recordings, transcripts and photos were electronically secured on the cloud with copies on a computer and an external hard drive. The physical forms are kept in a safe lock of repositories at the University of Ibadan, while the virtual data are stored in an encryption-protected cloud storage. Confidentiality was ensured by conducting the interviews separately and in settings where the participants felt safe and comfortable enough to speak freely. Anonymity has been ensured by redacting all identifiers, names and locations that can compromise the privacy of participants.

## Results

**Fig 1:**
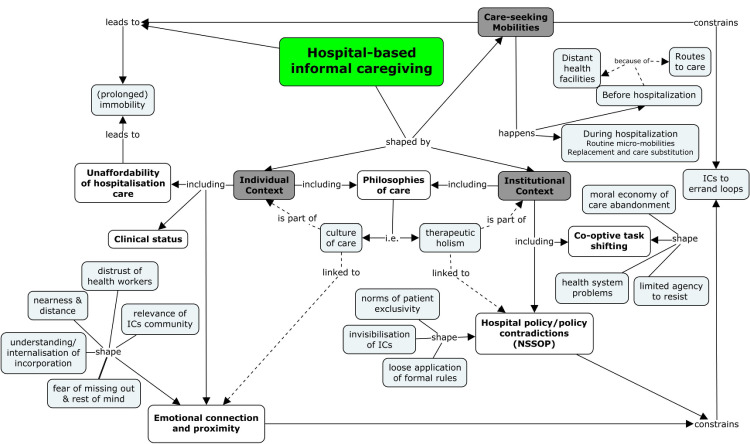
Hospital-based informal caregiving framework.

This section presents evidence showing the role of care mobilities and other contextual factors in hospital-based informal caregiving (HIC) in Nigeria (see Fig 1). Care mobilities, encompassing various forms of movements and routines, play a pivotal role in shaping the presence and activities of informal caregivers (ICs) within tertiary health facilities. This section presents the findings of our study, organized into two overarching themes: *care mobilities* and *associated contexts of hospital-based informal caregiving (HIC)*. The first theme, *care mobilities*, explores the physical and logistical movements undertaken by informal caregivers (ICs) before hospitalization including long-distance travel and during hospitalization involving routine micro-mobilities within the hospital environment. The second theme, *associated contexts*, delves into the multifaceted drivers of HIC, categorized into *individual*, *institutional*, and *cross-cutting* dimensions.

At the *individual level*, we examine how emotional connections, the clinical status of patients, and financial constraints shape caregivers’ involvement. The *institutional context* highlights policy contradictions and the co-optive task-shifting practices that normalize ICs’ roles despite formal exclusion. Finally, the *cross-cutting context* reveals how cultural norms and therapeutic holism intersect to reinforce the expectation of informal caregiving. The presentation of these themes is followed by a profound discourse on how they shape specifically, how mobilities influence caregivers’ activities, and more generally, HIC in (south-west) Nigeria.

### Care mobilities before and during hospitalization

Two sub-themes emerged before and during hospitalization such as long distance to the health care centres and routine micro mobilities.

#### Long distance to health care centres.

ICs navigate multiple healthcare centers with patients before reaching the tertiary hospital, often traveling from distant metropolitan areas, other states or provinces, and even regions beyond Southwestern Nigeria. As companions to sick relatives, ICs partake in care-seeking movements necessitated by challenges such as costly or impractical daily commuting, leading to their prolonged presence in or around the healthcare facility. For instance, a caregiver shared that:

*“…First of all, we went to Gwagwalada, from Gwagwalada, we went to the National Hospital, then from there we came down to Adamawa, we visited another huge hospital there in Adamawa too, then we came down to the East[ern Nigeria], we went to teaching hospital at Nnewi...”* (IDI 19/female/25)

The need for these mobilities was shaped by the necessity of seeking help for an illness, with serious implications for worsening the health of sick relatives. The participant further elaborated: *“…it’s a long distance and the expense, the money. …After the long journey, he breaks down and all that.”* (IDI 19/female/25).

#### Routine micro-mobilities.

Also, HIC often necessitates movement as part of supportive care practices. ICs engage in routine movements essential for providing supportive care, including running errands within and outside the hospital. These routine mobilities constitute a significant aspect of ICs’ activities. Errand tasks form a substantial portion of their daily activities, including sourcing water, both hot and normal temperature, primarily for the inpatient but also for personal use. ICs also engage in activities such as conducting investigations (assisting with taking blood and other samples to the lab) and purchasing food and medication, both within and outside the hospital premises. Furthermore, a pattern of routine “going and coming” emerges, characterized by frequent trips between the hospital and home, as well as short periods of (semi-)hospitalization to assist sick relatives. An inpatient illustrated this routine by describing her husband’s experience:

*“My husband has been with me, he goes…weekdays, he will go, weekend he will come [back]. But last weekend, he stays for days, since Saturday, Sunday, Monday, Tuesday, Wednesday he now left”* (IDI 31/female/38).

On the other hand, despite care giving involving a lot of routine mobilities, care mobilities often lead to immobility for ICs in some other cases. The experience of immobility is due to the inability to settle medical bills, resulting in the prolonged stay of both inpatients and ICs within the hospital premises. As a management staff of the hospital observed:

“*There is a situation that this particular patient was on the ward, the mother, the woman has been on admission for more than two weeks, they came from all the way from XXX city, and the woman [caregiver] was putting pressure for discharge, she said she has exhausted her leave, that she has to resume back to work*.” (KII 6/female/43)

#### Replacement and care substitution.

Our findings also highlight the possibility of occasional absence or impermanence among ICs. One approach employed by ICs is to leverage the tracking process implemented within the hospital. Nurses or other healthcare workers in need of ICs’ attention can reach them via mobile phone. Another method involves arranging for a replacement caregiver. This option is particularly favored by ICs traveling from beyond the city, as indicated by a security guard:

“*If another person is coming to replace [another IC], they will bring the replacement before the original caregiver leaves. They will say, ‘Oh! I’m going,’ although the replacement will stay to take care of my patient*” (KII 2/male/59).

Finding a replacement was easier for ICs who have other family members with them in the hospital. In very few cases, an inpatient may have more than one IC hanging around to support them. Conversely, those who did not have other family members’ present adopted other means of managing absence by planning their absence along with approved visiting hours of the hospital. Yet another approach was to arrange with a third-party, such as paid ad-hoc carers or a sympathetic staff of the hospital, especially health assistants. However, unlike ad-hoc carers who are paid a fixed amount for their time, staffers who stand-in temporarily were not particularly reliable, which may affect the quality of care given to the inpatient.

### Associated contexts of hospital-based informal caregiving

We further explore the nuanced landscape of HIC, shedding light on the diverse drivers of why relatives “stick around” to support hospitalization care for loved ones. These drivers have individual, institutional and cross-cutting dimensions. The individual level consists of sub-themes such as: emotional connection and proximity, clinical status of patient, and unaffordability of hospitalization while institutional dimensions manifest as policy contradiction in the practice of “non-standardised standard operating procedure” and what we call “co-optive task-shifting”. The prevailing philosophies of care in the hospital and larger society is crisscrossing at the intersection of individual and institutional contexts of HIC.

### Individual factors influencing HIC

#### Emotional connection and proximity.

The concept of proximity, such as, physical closeness in space, time, and relationship, emerged as a central theme in discussions with study participants. Our findings revealed that caregivers prioritize remaining close to their hospitalized relatives, driven by a deep sense of commitment and concern for their well-being. The emotional connection to inpatients, and the desire of ICs to remain close, is influenced by various factors such as their perception of distance, fear of missing critical moments, and need for peace of mind. Additionally, concerns about healthcare workers, the morale of both the inpatient and the ICs, and the perceived importance of being near other caregivers also play a role. Specifically, ICs’ perception of distance played a significant role in determining their presence near the hospital. For instance, one participant remarked, “...where I came from is far, I cannot be going and coming” (IDI 3/male/21), highlighting the practical difficulties of interstate or intercity travel. Some of them also expressed discomfort with staying elsewhere, as illustrated by one participant who said:

When I go to my friend’s [place], I will do vigil until morning, I can’t sleep. But when I am in here [in the hospital], even if I am on a seat and dozing, if I doze for 30 minutes, I am okay. Then I’ll start to pray. (IDI 15/female/72)

The quotation above indicates a reluctance to stay away from the hospital environment, even if alternative lodgings were available. This sentiment was similarly captured by one IC who raised concern about the emotional impact of staying away from the hospital:

One will not have rest of mind, to bring and leave someone in the hospital and return home to sleep. If it’s someone’s husband or wife, the home that they returned to sleep will not be different from sleeping outside. (IDI 10/female/25)

Their perception of proximity was also influenced by their fear of missing out and their need for peace of mind. They worried about being absent when critical events occur such as the deterioration or even death of an inpatient. Being near provided them with a sense of reassurance and tranquility, contrasting with feelings of restlessness when away from the hospital environment. For instance, a 72-year-old woman, whose daughter had been hospitalized for 21 days, expressed her reluctance to leave the hospital despite having friends in town. She stated:

*“...My friends encourage me to come but I tell them I am not coming… Maybe it’s because my daughter is here so my body is not at rest when I go elsewhere to sleep as compared to when I am in the ward, in the hospital environment; that is when my mind is at rest*.” (IDI 15/female/72).

In addition, ICs also cited a lack of trust in healthcare workers as a reason for remaining near their sick relatives. They perceived leaving while their relative was hospitalized as neglectful, equating it with risking the patient’s life. Furthermore, staying close was seen as a morale booster for both ICs and inpatients, providing emotional strength and support during the illness journey. The sense of community around the ward also played a role in their decision to stay nearby, although this aspect was not highlighted by majority (about 80%) participants. Overall, ICs’ perception of proximity was shaped by a complex interplay of emotional, practical, and social factors, underscoring the multifaceted nature of their caregiving experience.

#### Clinical status of inpatient.

The clinical condition of inpatients is a crucial factor in understanding why ICs provide HIC. Inpatients who are clinically unstable or in critical condition are most in need of extra support. Notably, a considerable portion of patients admitted through the emergency ward fall into this category of “critical status”.

In the study setting, hospitalization often involves a “settlement stage.” During this stage, various critical procedures take place, including patient clerking, life-saving interventions, stabilization measures, and crucial decision-making processes. These factors underscore the importance of having relatives present. As elucidated by a management staff member of the hospital:

*“…You know…when a patient that is critically ill, and, definitely, the patient has not really been settled down, he still, the patient, still requires you know some materials that will still be needed. It’s not the nurse, it’s not the doctor that will be running around for that patient…”* (KII 5/male/55)

Inpatients assigned to wards may also fall into the group as well, especially if they were considered “very ill” or living with “…terminal illness, or illness that is very severe, unconsciousness, stroke” (KII 1/male/50). Another management staff highlighted this point as follows:

…When there is incapacity in a patient, that is when you need the situation which you’re talking about; because this person cannot go and buy food, he cannot wash his clothes or change his clothes, he cannot do anything. So that’s where you have people accompanying to help, so in that situation it’s majorly for the elderly or for children. Or for those that are really very ill; terminal illness, or illness that is very severe, unconsciousness, stroke, all those things. (KII 1/male/50)

#### Unaffordability of hospitalization care.

Our findings revealed that ICs’ inability to afford the daily cost of hospital bed space and supportive services necessitates their presence. Hospitalization is financially burdensome for many low-income individuals referred to tertiary hospitals for specialized care. This economic strain extends to relatives accompanying the hospitalized, who often act as fundraisers on behalf of the patients.

While financial constraints may limit some family members’ ability to be present, those who can afford hospitalization costs have more flexibility. However, many ICs, like the patients they support, pay for hospitalization out of pocket and provide care materials for healthcare workers as needed. This was corroborated by some of the hospital staff, including a nurse who noted: *“…We told them that, ‘pay for drug deposits so that there will not be need to come in or be calling you from time to time for drugs.’ …But some of them, because of financial constraint, they will not be able to pay for it, …maybe the time you ask them to get the drugs they have to source for money to procure it*.” (KII 16/female/45).

Another nurse corroborated that:

*“The only major challenge is finance…. relatives even prefer if they are able to get everything they need financially for the patient; their movement will be less, then the patient can talk to them on phone if that person cannot come. But where they are just managing, no money, they will use the coming and going to show support to the patient you know”* (KII 19/female/55)

Thus, “sticking around” becomes an option readily adopted by poor ICs as a way to show care for the hospitalized, aligning with the Yoruba saying, “bi a ko ni owo, aasa la’ajo,” meaning “if one does not have money, they can show care at least.”

### Institutional context

#### Policy contradictions: Practicing “non-standardised standard operating procedure”.

In operation, “non-standardised standard operating procedure” (NSSOP) are unwritten rules constructed by hospital management/staff and adhered to by caregivers as part of the process of providing hospitalisation care and services. The existence of these informal “rules” alongside stated policies sometimes run at a parallel. For example, the health facility enforces a visitation policy dictating designated times for visitors to access inpatient wards. These time slots, typically one hour each in the morning and evening, are prominently displayed at ward entrances and apply to both regular visitors and ICs supporting inpatients. The visitation policy, construed as the standard required of the health facility, also expressly prohibits ICs from being inside or near the wards outside of these designated visiting hours, with no expectation for them to stay overnight with the hospitalized individual. This stringent stance is encapsulated by the assertion that “*the hospital policy is you can’t stay with your patient*” (KII 3/male/59). The “norm of patient exclusivity” placed patients as raison d’être of the hospital, with very limited consideration for ICs, because IC presence could also be “disruptive” of orthodox operations.

However, in addition to the formal rule in place, there was also a noticeable parallel informal rule existing side-by-side the formal one: a “non-standardised standard operating procedure” (NSSOP). Whether coming from near or far, the hospital and staff managing day-to-day operation expected “someone” to be present to help *their inpatient*. The management team sought to find out prior to admission that an inpatient had someone *on the ground* to run errands. As a doctor said:

…*Whenever we have patients coming in for surgery, I would ask them how many people are coming with you? If you say one, I’ll tell you why don’t you look for two*. (KII 12/male/36)

This is because post-surgical care can be intensive involving activities such as watching out for flatulence and constant monitoring for any changes in patient condition – tasks which are non-expert yet too time-intensive for the already over-burdened medical staff. By asking “Who is going to do that for the patient?”, the hospital established and operated a parallel system which kept ICs’ presence normalised despite the formal rule in place to keep them away. Thus, whereas NSSOP rendered ICs wanted and necessary for hospitalization care, formal policy framed them as “unwanted” or “unneeded”.

#### Co-optive task-shifting as responsiblilisation regime.

Institutionally, there was a “responsibilisation regime” operating in the healthcare facility. Responsibilisation in this context refers to a situation in which part of the care responsibilities for inpatients are informally transferred to their relatives; thus, causing tasks to be shifted to and co-performed by informal caregivers and hospital staff, despite a formal designation of such tasks to specific cadres of staff. Sharing and shifting these tasks overtime has informally instituted this “system” of doing things where ICs (co)perform some staff duties as demanded or delegated. The ICs also frequently report back to the hospital staff on the results of specific delegated tasks. The phenomenon was entrenched, pervasive and contributory to why ICs provide HIC. As a management staff noted, ICs do “…only all the responsibilities that we delegate to [them]” (KII 6/female/43). Standing out from the assertion are words like “responsibilities” and “delegate” and relatives who accompany inpatients were expected to “take over” certain roles once their relation was placed on admission.

This responsibilisation regime thrive in the health facility due to the prevailing moral burden built around inpatient abandonment, health system problems, like institutional failure and staff shortages, and the limited agency of relatives to resist their institutionally-normalised involvement in hospitalisation care. For one, management staff and ICs often invoked the “absence equals abandonment” belief to construct an expectation of family participation in HIC. The notion became apparent from the position of a top hospital staff who said “If they [ICs] are not there, …it’s like you have abandoned that patient there” (KII 5/male/55). By constructing absence as abandonment of sick relatives, ICs committed to staying with the hospitalised while hospital staff expected and demanded it.

Apart from how absence (or presence) of IC was constructed, health system failure in Nigeria, as manifest in staff shortage, created a caregiving vacuum that relatives were constrained to fill. The reality of staff shortage especially was acknowledged as a challenge, particularly among nurses and the lowest cadre who typically performed many of the tasks that ICs have been coopted to perform. Therefore, ICs, according to a management staff:

…Are filling the gap because…the patient will need something at one point or the other. So, and if either a carer or a relation is not on ground, there is nobody that will handle that [task]. (KII 5/male/55)

Surely, unfilled operational vacuum has been disruptive to the smooth running of hospital services. To push trolleys as an example, “…one person is not enough to move the trolley, so they always want a relative to be on ground to assist…” (KII 26/female/36). Similarly, care might be stopped if a patient did not have a relative on the ground. Taken together, the operational vacuum in place and the shared belief that “absence equals abandonment” made the shifting of care responsibilities to ICs meaningful.

In the operation of co-optive task-shifting, however, ICs have limited agency to resist their incorporation, even when they were conscious that they might be “doing a little too much”. An elderly woman who was caring for her husband hinted at her awareness of “doing too much”, observing that:

The patient that can’t stand up… Am I the one that is supposed to carry him to the toilet? …Am I the one that is supposed to carry bed pan to the patient? (IDI 7/female/65)

Still, she was attentive to the danger of her refusal to participate in the care of her husband. For this participant especially, she did not resist her co-option into performing these tasks because she feared that her husband will be maltreated – her husband actually reported to her that he was maltreated. The maltreatment story bred distrust, such that ICs endeavour to be close and not leave their relatives in care of health workers completely.

Finally, the operation of responsibilisation led ICs to believe that, by hanging around, they were taking responsibilities off their relatives. As noted below:

It’s like you are playing with the life of your patient if you do not stay with them. It is better to leave them at home than bring them to the hospital if you know you will not stay. (IDI/female/25).

Put differently, because of the normalisation of their role, ICs tended to internalize the idea that they are responsible for their relatives, such as when an IDI participant (female/65) said, “they [health workers] cannot do the work I will do… They have their own duty; I have my own duty to perform as a caregiver.” Such internalization of informal caregiving role tend to freed hospital health workers from taking full responsibility for the care of the sick, thereby by aiding ICs’ co-option.

### Complementary philosophies of care as a cross-cutting context of HIC

At the intersection of individual and institutional contexts driving the practice of HIC is the reality of two complementary philosophies of care, that is culture and therapeutic holism. These care philosophies operate side-by-side to influence the presence of informal caregivers (ICs) during their relatives’ hospitalization. The enduring culture of care, deeply rooted in many African societies, emphasizes reciprocal kinship relations and the shared responsibility of caregiving across generations, which directly feeds into therapeutic holism, a concept that argues total wellness for a patient can only be achieved by a collaboration of professional and familial care. Thus, therapeutic holism requires the presence of family carer as role-played by informal caregivers, giving room for the cultural expression of care as well.

For ICs, the need to be present is viewed as an unavoidable duty, as reflected in the Yoruba proverbial saying, “kii de bani ka yeri,” meaning “one does not run away from one’s challenges” (IDI 1/female/55). This is rooted in the cultural belief that everyone must face a period of trial and when it comes, one should be ready to carry the cross. Reflecting this belief on caregivers’ strain, it appears that though HICs face strenuous burdens, it is *their* burden. Thus, the burden of hospitalization and its associated challenges are not solely borne by the inpatient. Also, various categories of hospital staff acknowledged the cultural expectation of informal caregiving. For instance, a medical doctor noted, “...we are still a largely community-based people; it will take us a while to not need people around for health care needs” (KII 25/male/36). Within this cultural framework, providing HIC is viewed as an act of love and obligation. However, it appears that not all individual is so embracing of this cultural framework. For one 24-year-old HIC caring for an uncle which he supposedly barely knows, his presence with his uncle-patient was involuntary, and if he had his way, he would rather “…be at school, reading or hanging out with friends instead of being stuck here going through all this stress.” He was the only one who expressed this resistance. For him, though the cultural philosophy did not yield any acceptance of the caregiving burden, the cultural compulsion of obeying one’s parents did, as he was mandated by his father to stay with his uncle. And to this instruction, he could not say no.

In addition to culture, therapeutic holism emerged as another guiding principle shaping ICs’ presence. Unlike culture, which reflects broader societal norm that individuals internalize, in this case, as being support mechanisms for one another, therapeutic holism focuses on the involvement of family members in patient care by the hospital to optimize clinical outcomes. Healthcare workers, primarily nurses but also doctors, mentioned therapeutic holism as a key motivator for requiring ICs’ presence. Beyond routine assistance, involving ICs in patient care is seen as an opportunity to prepare them for post-discharge responsibilities. Nurses may invite ICs to participate in procedures that will continue post-discharge, such as managing a nasogastric tube (KII/female/45). Doctors also encourage ICs to observe and cooperate in achieving comprehensive care for the patient.

## Discussion

This article investigates the impact of care mobilities, as well as other contextual factors, on why and how relatives of inpatients engage in hospital-based informal caregiving. It introduces an innovative framework of caregiving mobilities to understand HIC by shedding light on the shared experience of care-seeking journeys undertaken by patients seeking specialized treatment in Nigerian tertiary health facilities. Care mobilities and the notion of distance embedded within it goes beyond accessibility to healthcare facilities [38] and the choice of patients and their ICs [39]. It also highlights the rural-urban divide that have become prominent in health accessibility conversations in low- and middle-income countries [40,41].

Currently, migration health and public and population health research rarely adopt the mobilities lens nor do they pay attention to ICs who often accompany the sick on their journey to find solution to ill-health. This study filles in the gap by introducing an innovative framework that bridges mobilities studies with health systems research, offering a novel lens to examine care-seeking dynamics in low-resource settings. By highlighting the “mobile condition of care-seeking”, this framework reveals how the physical, social, and economic journeys of patients and their informal caregivers (ICs) shape health outcomes and system functionality. Its novelty lies in challenging fixed ideas of healthcare access by showing how care moves across households, communities, and institutions within health systems. This approach uncovers critical, yet understudied, actors like ICs, whose labour and sacrifices sustain care processes but remain marginalized in policy.

At the centre of this framework is the redefinition of “distance” as a collective burden. While geographic barriers to healthcare in low- and middle-income countries (LMICs) are well-documented [42], what is rarely represented is how distance is vicariously experienced by ICs through financial strain, emotional stress, and physical exhaustion. The article shows how distance is equally a problem for family members who experience the negative consequences of distance while remaining marginal to the health system structure. This implies that there is the need to integrate the experiences of ICs into health policies and interventions, as their emotional, social, and economic burdens are deeply tied to the accessibility and quality of care for the patients they support. This calls for a more comprehensive approach that addresses the broader implications of care mobilities in health systems research.

Further, findings also draw attention to the structure of informality that shapes care provision in an under-resourced setting. Here, the formal care practice of the hospital and the everyday standard operating procedure that healthcare staff work by depended a lot on the presence of ICs [11]. The presence and activities of ICs immensely shapes this informality which creates a distinct social world in the hospital setting considerably characterized by complex relationships between staff and ICs [43]. The presence of ICs was assumed and expected when a patient is brought in and admitted to the wards. Strikingly, however, the entrenched practice of the NSSOP did not translate to a qualitative inclusion of ICs in the health facility. Rather, ICs remained excluded as the norm of “patient-exclusivity” characterizes institutional disposition [13]. Patience-exclusivity made the policy that prohibited the presence of ICs reasonable and valid. It is also within the logic of this institutional disposition that the hospital seemingly invisibilize ICs, despite their visible presence in the healthcare facility always. Here, another tension is revealed between the norm of patient exclusivity and narrative of therapeutic holism which paradoxically also functions to facilitate the co-optive incorporation of ICs in an Africa tertiary health facility. These dynamics reveal the need for a more inclusive approach to healthcare that acknowledges and integrates the vital role of ICs in the provision of care.

Relatedly, the practice of NSSOP is suggestive of the problems arising from co-optive task shifting as part of an oppressive health systems transition that is underway in Africa [1]. While recognising the ongoing crisis of staff shortage and the usefulness of engaging ICs to fill the vacuum in some ways, the existing operation of task-shifting is worth reassessing. When a management staff says, they give “responsibilities” and “delegate” to ICs, they are redefining the domain of duties within a system in which the bureaucracy inherent in everyday operation of the hospital creates diverse opportunities for routine tasks. The tasks so created are such that those formally employed staff are unable to perform them alone and effectively without the contribution of ICs. Interestingly, the World Health Organisation (2008) describes task-shifting as the process of rationally delegating tasks to “less specialised health workers” with shorter training and fewer qualifications to improve access to healthcare services. However, the role being performed by ICs in Nigeria tertiary health facility is an example of this delegation, although most of them lack formal care training in the sense intended by the WHO. The ICs involved in HIC often assumed their roles suddenly and unprepared.

Moreover, by paying attention to how nurses interact and engage the labors of caregivers of inpatients, we can begin to understand the utility/use mode of incorporation of family caregivers in African public health facilities. Although many relatives of inpatients may assist loved ones on accounts of what Sahlins [44] called “generalized reciprocity”, the utilization of the labor in the health facility was normalized, and ICs have limited power to determine how their labor is utilized. While not expecting anything from their sick loved ones, the formal caregivers often activate and de-activate the labor of ICs at will. Formal health workers may dismiss and mobilize the labors of family caregivers, and determine whether they are wanted or not wanted as desired. In short, ICs have little or no power to challenge formally employed staff, and have become a path of least resistance exploited to sustain a dysfunctional health system. This evidence further contributes to our understanding of the undercurrents of unequal power between ICs and health workers in African public health systems [45,46].

Meanwhile, the constraining of ICs to hang around as supported by the notion of “absence equals abandonment” ignores the changing pattern of social relations in many African societies today, where increasingly people are moving towards individualism and small families – away from communalism and extended family structure [47, 48]. This norm also ignores the damaging economics of presence on poor people who mostly attend such public hospitals; people who mostly work in the informal economy as day-to-day earners [49–51]. When stuck in the hospital, they risk losing livelihoods and income. They are also unlikely to be able to afford the catastrophic expenses associated with supporting the hospitalization care of sick relatives [52–54]. This highlights the need for a more flexible and economically sensitive approach to caregiving in public hospitals, one that recognizes the social and financial constraints ICs face and offers alternatives that do not jeopardize their livelihoods. An example of this could be the introduction of caregiver stipends or compensation which has been documented in Canada and the United Kingdom [55].

This explanatory framework underscores the importance of relational sociological theories in healthcare, particularly concerning informal caregiving, including social capital, network and health inequalities theories [56–59]. For example, the framework aligns with Coleman’s social capital theory by highlighting the significance of relationships between and among actors, rather than focusing solely on individual attributes. It suggests that support from relatives is influenced by social capital within families and communities, especially strong intergenerational ties and trust, which can facilitate support during hospitalization, despite potential challenges. We can advance similar perspective in relation to network theory [60]. The notion of care mobilities also shows how health services access and distance-related issues worsens health inequalities, particularly for ICs and inpatients from lower socioeconomic backgrounds. Due to the necessities of care mobilities, they may have less flexibility in taking time off from work, arranging transportation, or accessing information about the patient’s condition. Our framework, therefore, reiterates that policies that address the root causes of health inequalities, such as income inequality and social exclusion, can empower relatives to provide optimal support to their loved ones during hospitalization.

This study’s findings demand concrete policy interventions addressing the exploitative power dynamics between healthcare workers (HCWs) and Informal Caregivers (ICs), who endure the collective burden of distance through financial strain, emotional stress, and physical exhaustion while remaining marginal within the system. To improve support for informal caregivers (ICs) and address healthcare inequities, three targeted policy actions are recommended. First, the government should prioritize investments in healthcare infrastructure and expand mobile and community-based primary health services in rural and underserved urban areas. For example, deploying mobile clinics or community health workers can reduce travel burdens and increase service uptake. Second, healthcare institutions must adopt patient- and caregiver centered models of care by formally recognizing the caregiving role of ICs in treatment planning. This includes integrating culturally relevant practices and training healthcare professionals in culturally competent communication and care delivery. Third, financial protection mechanisms should be introduced to reduce out-of-pocket expenses for ICs. These could include means-tested subsidies, community-based health insurance, and voucher systems to cover hospitalization, medications, and transport.

These policy actions must be grounded in partnerships among federal and state governments, health agencies, NGOs, and local communities to ensure they are contextually appropriate and sustainable.

### Limitations of the study

While the study advances an explanatory framework for why relatives inpatients provide hospital-based informal caregiving, data was collected from only one health facility in Southwestern Nigeria. By implication, the researchers had access to only a limited access to potential participants and we could not explore the very diverse contextual factors that may vary how HIC is performed in other parts of Nigeria. Therefore, the findings should be interpreted with caution. Nonetheless, the study is valuable and relevant as it attempted to systematically address an underexplored aspect of informal caring, thereby providing a foundation for future research in the area.

## Conclusion

The role of informal caregivers remains pivotal within the healthcare landscape of low-resource settings. This is particularly important in the context of hospitalization care, where patients often travel long distances to access specialized treatment in urban tertiary health facilities. Understanding the factors that shape the involvement of these caregivers is crucial for optimizing patient care outcomes. This study offers empirically-informed insights and a framework that go beyond the traditional understanding of the culture of care in African societies.

Our findings present different opportunities for further research. First, there is a need for more in-depth qualitative and quantitative studies to explore the lived experiences of ICs across different cultural and geographical contexts within Africa. Such research could provide a more nuanced understanding of the socio-economic and emotional burdens faced by ICs and how these vary across different settings. Additionally, future studies could investigate the feasibility of interventions such as caregiver stipends, community-based care programs, and training for healthcare professionals on culturally competent care in Africa.

Second, the concept of care mobilities warrants further exploration, particularly in relation to its impact on health outcomes and the sustainability of health systems. Research could examine how care mobilities intersect with other determinants of health, such as gender, socio-economic status, and urban-rural disparities, to shape access to and quality of care. Comparative studies across different countries could also shed light on how care mobilities operate in diverse health systems and what lessons can be learned from successful models in other regions.

Third, the role of technology in mitigating the challenges of care mobilities and supporting ICs should be explored. For instance, telemedicine and mobile health applications could potentially reduce the need for physical travel to healthcare facilities and provide remote support for ICs. Research could assess the feasibility, acceptability, and impact of such technologies in low-resource settings.

Finally, there is a need for policy-oriented research that evaluates the implementation and impact of healthcare reforms aimed at integrating ICs into the formal health system. This could include studies on the development of national caregiver support policies, the establishment of caregiver training programs, and the creation of financial mechanisms to alleviate the economic burden on ICs. Such research would be crucial in informing evidence-based policies that promote the well-being of both patients and their caregivers.

Moving forward, it is imperative for future research to broaden the scope of investigation into hospital-based informal caregiving across diverse African contexts. Additionally, comparative studies exploring how the findings from this research may translate to non-African settings could provide valuable insights. Furthermore, there is a need for deeper exploration into the experiences of hospitalized patients who lack familial support within health systems where the role of informal caregivers is deeply ingrained. By exploring these areas, we can advance understanding on the complexities surrounding informal caregiving in resource-limited health systems settings.

## Supporting information

S1 Table Socio-demographic information: informal caregivers and hospitalised patients. (DOCX)

S2 Table Socio-demographic information: key informants. (DOCX)

S1 File Inclusivity in global research. (DOCX)
