# Supplementary material for: Care mobilities and associated contexts of hospital-based informal caregiving in Nigeria: Towards an explanatory framework
Source: PLoS One. 2025 Jul 31;20(7):e0327198. doi: 10.1371/journal.pone.0327198 (PMC12313061; doi:10.1371/journal.pone.0327198)
Supplement: S2 Table — (DOCX) [file pone.0327198.s002.docx]

**Supplementary File**

Table 2: Socio-demographic information: Informal caregivers and hospitalised patients

| Variable | Category | Informal caregivers (*N*=21) | | Hospitalised patient (*N*=15) | |
| --- | --- | --- | --- | --- | --- |
|  |  | Frequency | Percent | Frequency | Percent |
| Gender | Female | 15 | 71% | 9 | 60% |
|  | Male | 6 | 29% | 6 | 40% |
| Age | 18-24 | 3 | 14% | 3 | 20% |
|  | 25-34 | 2 | 10% | 3 | 20% |
|  | 35-44 | 6 | 29% | 4 | 27% |
|  | 45-54 | 3 | 14% | 2 | 13% |
|  | 55-64 | 4 | 19% | 1 | 7% |
|  | 65+ | 3 | 14% | 2 | 13% |
| Education | Primary | 2 | 10% | 3 | 20% |
|  | Secondary | 9 | 43% | 3 | 20% |
|  | Tertiary | 8 | 38% | 8 | 53% |
|  | Not provided | 2 | 10% | 1 | 7% |
| Ethnicity | Yoruba | 16 | 76% | 12 | 80% |
|  | Others | 5 | 24% | 3 | 20% |
| Marital status | Single | 4 | 19% | 4 | 27% |
|  | Married | 16 | 76% | 11 | 73% |
|  | Not provided | 1 | 5% | - | - |
| Religion | Islam | 6 | 29% | 3 | 20% |
|  | Christianity | 14 | 67% | 12 | 80% |
|  | Not provided | 1 | 5% | - | - |
|  | *Total* |  |  |  |  |
| Family type | Nuclear | 14 | 67% | 14 | 93% |
|  | Extended | 5 | 24% | 1 | 7% |
|  | Single parent | 1 | 5% | - | - |
|  | Not provided | 1 | 5% | - | - |
| Occupation | Informally employed | 14 | 67% | 4 | 27% |
|  | Formally employed | 1 | 5% | 6 | 40% |
|  | Student | 1 | 5% | 2 | 13% |
|  | Retiree | 4 | 19% | 1 | 7% |
|  | Others | 1 | 5% | - | - |
|  | Unemployed | - | - | 2 | 13% |
| Monthly income | 5000-10000 | 3 | 14% | 1 | 7% |
|  | 10001-20000 | 2 | 10% | 0 | 0% |
|  | 20001-50000 | 3 | 14% | 4 | 27% |
|  | 50001-100000 | 3 | 14% | 1 | 7% |
|  | 100001-150000 | 3 | 14% | 2 | 13% |
|  | 150000+ | 1 | 5% | - | - |
|  | Not provided | 6 | 29% | 7 | 47% |
| Patient/caregiver gender | Female | 10 | 48% | 11 | 73% |
|  | Male | 11 | 52% | 4 | 27% |
| Patient/caregiver age | Below 18 | 1 | 5% | - | - |
|  | 18-24 | 3 | 14% | 2 | 13% |
|  | 25-34 | 3 | 14% | 3 | 20% |
|  | 35-44 | 4 | 19% | 1 | 7% |
|  | 45-54 | 3 | 14% | 3 | 20% |
|  | 55-64 | 3 | 14% | 2 | 13% |
|  | 65+ | 4 | 19% | 3 | 20% |
|  | Not provided | - | - | 1 | 7% |
| Caregiver-patient relationship | Parent | 10 | 48% | 3 | 20% |
|  | Spouse | 4 | 19% | 7 | 47% |
|  | Sibling | 5 | 24% | 2 | 13% |
|  | Children | - | - | 1 | 7% |
|  | Other relatives | 2 | 10% | 1 | 7% |
|  | Co-worker |  |  | 1 | 7% |
| Place of residence | In-state | 8 | 38% | 2 | 13% |
|  | Out-state | 13 | 62% | 13 | 87% |
| Duration of stay (weeks) | Less than 1 | 3 | 14% | 4 | 27% |
|  | 1-2 | 8 | 38% | 3 | 20% |
|  | 3-4 | 5 | 24% | 4 | 27% |
|  | 5+ | 5 | 24% | 4 | 27% |
| Ward | Medicine | 5 | 24% | 1 | 7% |
|  | Medicine (oncology) | - | - | 1 | 7% |
|  | Medicine (urology, diabetes) | 3 | 14% |  |  |
|  | Obstetrics and gynaecology | 3 | 14% | 3 | 20% |
|  | Orthopaedic | 1 | 5% | - | - |
|  | Paediatrics | 1 | 5% | - | - |
|  | Surgery | 4 | 19% | 4 | 27% |
|  | Surgery (oncology) | 4 | 19% | 4 | 27% |
|  | Surgery (orthopaedic) | - | - | 2 | 13% |
| Paired interviews | No | 12 | 57% | 6 | 40% |
|  | Yes | 9 | 43% | 9 | 60% |

Table 3: Socio-demographic information: Key informants

| Variable | Category | Frequency (N=36) | Percent (100 %) |
| --- | --- | --- | --- |
| Gender | Female | 27 | 60% |
|  | Male | 9 | 40% |
| Age | 18-24 | 1 | 3% |
|  | 25-34 | 6 | 17% |
|  | 35-44 | 14 | 39% |
|  | 45-54 | 10 | 28% |
|  | 55-64 | 4 | 11% |
|  | Not provided | 1 | 3% |
| Education | Secondary | 6 | 17% |
|  | Tertiary | 18 | 50% |
|  | Fellowship | 6 | 17% |
|  | Masters | 5 | 14% |
|  | PhD | 1 | 3% |
| Ethnicity | Edo | 1 | 3% |
|  | Igbo | 6 | 17% |
|  | Isoko | 1 | 3% |
|  | Yoruba | 27 | 75% |
|  | Not provided | 1 | 3% |
| Marital status | Single | 5 | 14% |
|  | Married | 30 | 83% |
|  | Divorced | 1 | 3% |
| Religion | Islam | 5 | 14% |
|  | Christianity | 30 | 83% |
|  | Not provided | 1 | 3% |
| Hospital staff category | Health assistant (environment) | 5 | 14% |
|  | Health assistant (ward) | 5 | 14% |
|  | Doctor | 5 | 14% |
|  | Management | 6 | 17% |
|  | Nurse | 10 | 28% |
|  | Security | 5 | 14% |
| Ward/unit | Medicine | 3 | 8% |
|  | Medicine (urology) | 2 | 6% |
|  | Obstetrics and gynaecology | 1 | 3% |
|  | Orthopaedic trauma | 1 | 3% |
|  | Paediatrics | 2 | 6% |
|  | Surgery | 4 | 11% |
|  | Surgery (oncology) | 2 | 6% |
|  | Surgery (orthopaedic) | 2 | 6% |
|  | Psychiatry | 4 | 11% |
|  | Others* | 15 | 42% |

*Other ward/unit of the hospital that key informants were drawn from included ENT, physiotherapy, private ward, special obstetrics and neonate sections. The staff interviewed also included those attached to key units of the hospital, such as social work, security, and other strategic departments.
